# Real-Time monitoring of intracellular wax ester metabolism

**DOI:** 10.1186/1475-2859-10-75

**Published:** 2011-09-30

**Authors:** Suvi Santala, Elena Efimova, Matti Karp, Ville Santala

**Affiliations:** 1 Department of Chemistry and Bioengineering, Tampere University of Technology, Korkeakoulunkatu 8, FI-33720 Tampere, Finland

**Keywords:** wax ester metabolism, *Acinetobacter baylyi *ADP1, long chain aldehyde, bacterial luciferase

## Abstract

**Background:**

Wax esters are industrially relevant molecules exploited in several applications of oleochemistry and food industry. At the moment, the production processes mostly rely on chemical synthesis from rather expensive starting materials, and therefore solutions are sought from biotechnology. Bacterial wax esters are attractive alternatives, and especially the wax ester metabolism of *Acinetobacter *sp. has been extensively studied. However, the lack of suitable tools for rapid and simple monitoring of wax ester metabolism *in vivo *has partly restricted the screening and analyses of potential hosts and optimal conditions.

**Results:**

Based on sensitive and specific detection of intracellular long-chain aldehydes, specific intermediates of wax ester synthesis, bacterial luciferase (LuxAB) was exploited in studying the wax ester metabolism in *Acinetobacter baylyi *ADP1. Luminescence was detected in the cultivation of the strain producing wax esters, and the changes in signal levels could be linked to corresponding cell growth and wax ester synthesis phases.

**Conclusions:**

The monitoring system showed correlation between wax ester synthesis pattern and luminescent signal. The system shows potential for real-time screening purposes and studies on bacterial wax esters, revealing new aspects to dynamics and role of wax ester metabolism in bacteria.

## Background

Wax esters (WE) are oxoesters of long-chain fatty acids esterified with long-chain alcohols. WEs are industrially valuable lipid compounds exploited in several purposes including cosmetics, candles, printing inks, lubricants, surface coatings, and food industry. Today, WEs are mainly produced chemically or in enzyme reactors from rather expensive raw materials, and thus there is a strong need for biological production of inexpensive jojoba-like wax esters from renewable resources [1]. Naturally existing wax esters are commonly found e.g. in plant epicuticle, beeswax and *Spermaceti *oil of sperm whales. Among bacteria, wax esters are frequently produced by several *Acinetobacter *species for a carbon storage material [2]. The chemical structure of these waxes are similar to those found for example in jojoba (*Simmondsia chinensis L*.), thus making the bacteria an attractive alternative for producing wax esters by biotechnological means [3]. Furthermore, the wax ester composition can be altered by varying the substrate or growth conditions [3].

The biochemical pathway for wax ester synthesis in *Acinetobacter *sp. involves three enzymatic steps; in the first step, the acyl coenzyme A (acyl-CoA) is reduced to the corresponding aldehyde, a specific intermediate in wax ester synthesis, by a NADPH dependent acyl-CoA reductase [4]. The long-chain aldehyde is further reduced to the corresponding alcohol by an aldehyde reductase. For *Acinetobacter*, however, this enzyme is yet uncharacterized. In the last step, the fatty alcohol is esterified with a fatty acyl-CoA by an acyl-CoA:fatty alcohol acyltransferase (wax ester synthase) of a bifunctional enzyme WS/DGAT, leading to the formation of a wax ester molecule. This highly unspecific enzyme, originally characterized from *Acinetobacter baylyi *ADP1 by Kalscheuer *et al*. [5], has been utilized in various studies regarding the production of bacterial storage lipids, and suggested as a potential biocatalyst for WE production from long-chain alcohols and fatty acids *in vitro *and *in vivo *[6,7].

In order to exploit bacterial WEs industrially, more information is required for enabling the targeted enhancements of the natural production rates. According to conventional lipid analyses carried out by gas chromatography and thin layer chromatography, some conclusions can be drawn regarding WE production and accumulation in different hosts and at variable conditions. However, these methods are laborious and time consuming, and are not convenient for high through-put studies. An alternative method for neutral lipid detection based on fluorescence signal measurement has been introduced, but the method suffers from low specificity and sensitivity, and is thus not suitable for analysing WE specifically [8,9]. The lack of simple and rapid tools, based on e.g. reporter genes, for studying and monitoring the WE metabolism *in vivo *has restricted the amount of information obtained regarding the WE pathway dynamics. Bacterial luciferases (LuxAB) are luminescent proteins which utilize long-chain aldehydes as a substrate. The reporter genes *luxAB *encoding this enzyme have been widely exploited in biosensing applications, in which external substrate addition and a specific artificial regulation system is required [10]. In this study, by contrast, LuxAB was used for detection of intracellular substrate without addition of external substrate or regulation system. The potentiality of exploiting the WE intermediate utilizing LuxAB from *Photorhabdus luminescens *for monitoring WE metabolism in the natural WE producing host, *A. baylyi *ADP1, was investigated.

## Results

### Strain construction

**Figure 1:**
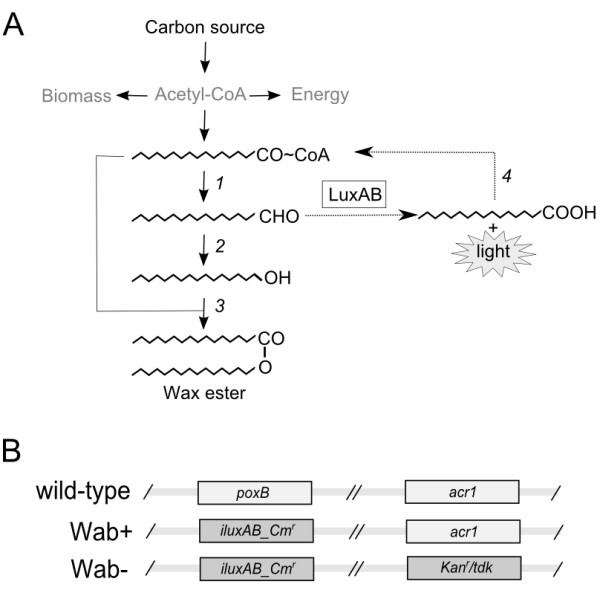
**Operating principle of the wax ester monitoring system in engineered *A. baylyi *ADP1**. A) The bacterial luciferace LuxAB utilizes the specific intermediate in wax ester synthesis route, fatty aldehyde, as a substrate producing light and fatty acid molecule which is returned to the carbon cycle. Enzymatic steps involved in the natural lipid metabolism of ADP1 are as follows: 1 - Fatty acyl-CoA is reduced to corresponding fatty aldehyde by the fatty acyl-CoA reductase (*acr1)*; 2 - Fatty aldehyde is reduced to corresponding fatty alcohol by an aldehyde reductase; 3 - Fatty alcohol is esterified with fatty acyl-CoA by WS/DGAT (*wax-dgaT*) forming a wax ester; 4 - Free fatty acid is recycled by acyl-CoA synthetases. B) A schematic presentation of the engineered strains Wab+ and Wab- against the wild type genotype. The gene cassette *iluxAB_Cm^r ^*containing the genes *luxAB *and chloramphenicol resistance under constitutive promoter was used to replace a neutral gene *poxB*. In Wab-, *acr1 *is replaced with a gene cassette *Kan^r^/tdk *[16].

In order to study the WE metabolism in *A. baylyi *ADP1, a synthetic gene cassette *iluxAB_Cm^r ^*containing bacterial luciferace *luxAB *was constructed using well-characterized biocomponents: a constitutive strong promoter (lac/T5), transcription termination loop (t lpp), selection marker (Cm^r^), and homologous sequences from ADP1 (downstream and upstream of the gene pyruvate decarboxylase *poxB*, ACIAD3381). The gene knock-out of *poxB *has been shown not to negatively affect the WE production or growth in *A. baylyi *ADP1 [11] and was thus used as a target site for gene replacement. The strain ADP1 wild type was transformed with the gene cassette and selected on LA plate containing 50 μg/ml chloramphenicol. The resulting strain ADP1Δ*poxB*::i*luxAB_Cm^r ^*was designated as Wab+. For controlling the specificity of the WE monitoring system in ADP1, a knock-out mutant lacking the gene for fatty aldehyde synthesis, fatty acyl-CoA reductase *acr1 *(ACIAD3383), was transformed with the same gene cassette and used as a control, since the mutant strain is known to be incapable of wax ester synthesis [4]. The resulting strain ADP1Δ*poxB::*i*luxAB_Cm^r^Δacr1*::*Kan^r^*/*tdk *was designated as Wab-. Also, for neglecting any background signal coming from the genetic construct, the gene cassette was cloned to plasmid and transformed to *E. coli *as an additional control. The WE synthesis pathway of ADP1 with the insertion of LuxAB is presented in Figure 1A, and a schematic illustration of the genetic differences between the engineered strains is shown in Figure 1B.

### Monitoring the WE synthesis in ADP1

**Figure 2:**
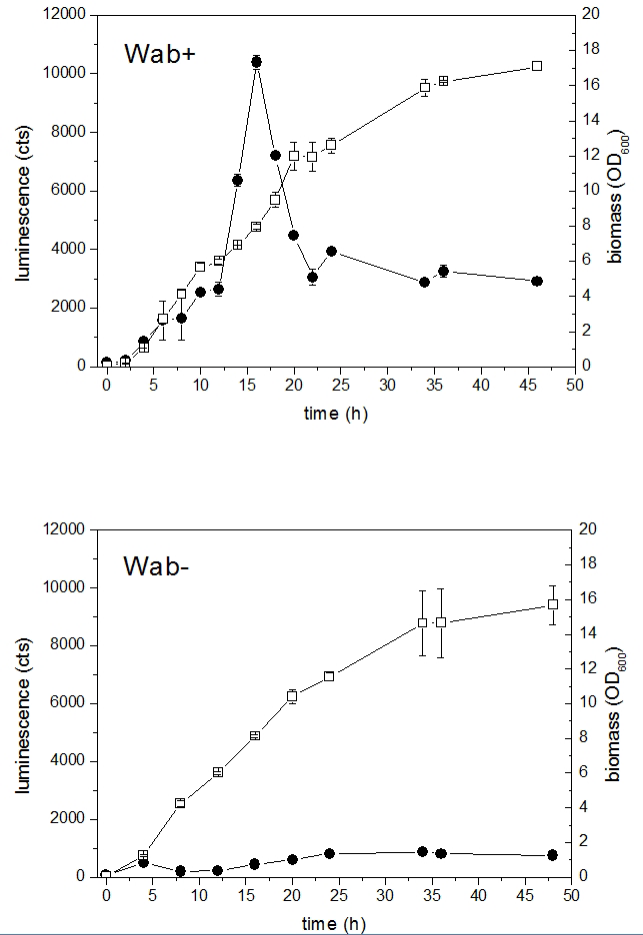
**Growth curves and luminescence of Wab+ and Wab- strains**. Wax ester metabolism was monitored by bioluminescence (cts, filled symbols) during growth (OD_600_, open symbols).

For monitoring the WE synthesis in ADP1, up to 46-hour batch cultivations were carried out for the constructed strains Wab+ and Wab-, using minimal salts medium supplemented with glucose. For *E. coli *expressing *luxAB *in the same culture conditions, no detectable luminescent signal was observed at any state of the cultivation, as shown also in other studies on different hosts [12], thus eliminating the possibility for any background signal coming from the synthetic gene construct. For the strains Wab+ and Wab-, the cell growth and luminescence signals are presented in Figure 2. For Wab+, the highest signal was measured between 13 - 16 h, after which the signal remained constant until the end of the cultivation. The cells reached stationary growth phase after 30 - 34 h of cultivation with optical density at wavelength (OD_600_) being approximately 16. For Wab-, low luminescent signal was measured throughout the cultivation, indicating some cellular activity towards long-chain aldehyde synthesis despite the gene deletion. However, this constantly low signal is not related to wax ester formation, as demonstrated by lipid analyses, and was thus considered here as a cellular background for ADP1. Total lipid contents and WE concentrations of Wab+ and Wab- cultivations were measured after 12, 34, and 46 h (Table 1) for extracted lipids. The wax ester concentration in the cultivations was analyzed by nuclear magnetic resonance spectroscopy (NMR) according to the determination of the ester bond concentration specific to WE.

**Table 1:** Total lipid and wax ester contents of Wab+ and Wab-strains.

	12 h	34 h	46 h
**Wab+**			
Total lipids (mg/l)	6.5	7.8	5.3
WE (μmol/mg lipids)	0.5	0.8	0.4
WE (μmol/l)	80.5	156	54.3
**Wab-**			
Total lipids (mg/l)	NA	5.2	3.7
WE (μmol/mg lipids)	NA	0	0
WE (μmol/l)	NA	0	0

NA - not analysed

WE - wax ester

According to NMR results, the WE content increased in the cells reaching the highest measured concentration at 34 h (156 μmol/l), after which both the total lipid and WE content were observed to decrease, at the end of cultivation the WE concentration being approximately 60% less (54.3 μmol/l). Furthermore, the proportion of WE from total lipids varied during the cultivation, the highest proportion (0.80 μmol/mg extracted lipids) being measured at 34 h. In the stationary growth phase of Wab+, the proportion of WE decreased faster compared to the amount of total lipids. For a control cultivation performed for wild type ADP1in identical conditions, similar pattern in WE accumulation and degradation was observed (data not shown). For Wab-, the wax ester content was zero at 34 h and 46 h, as expected. Also, the total lipid content was found to be smaller for Wab- compared to Wab+. Traces of free alcohols were detected for both strains, with no significant variation of the concentrations in terms of growth phase (data not shown). According to OD_600 _value, similar growth patterns were observed for both the strains for the first ~24 h, after which Wab- showed slight decrease in growth rate compared to Wab+. For monitoring the cellular luminescent protein level and the stability and functionality of the genetic construct in both Wab+ and Wab-, decanal was added to culture samples after 12, 24 and 46 h, resulting in prominent luminescent signals with equal magnitude regardless of the growth phase or strain (data not shown).

In order to determine the correlation between WE metabolism and carbon utilization, glucose concentration in the medium was measured after 12, 24, 34 and 46 h by high performance liquid chromatography (HPLC), the initial glucose concentration being 110 mM. The glucose consumption of Wab+ was found to be steady throughout the cultivation, and after 46 h a glucose concentration 1 mM was measured. For Wab-, glucose consumption was determined as an end-point measurement at 46 h, the glucose concentration being slightly higher, 8 mM.

**Figure 3:**
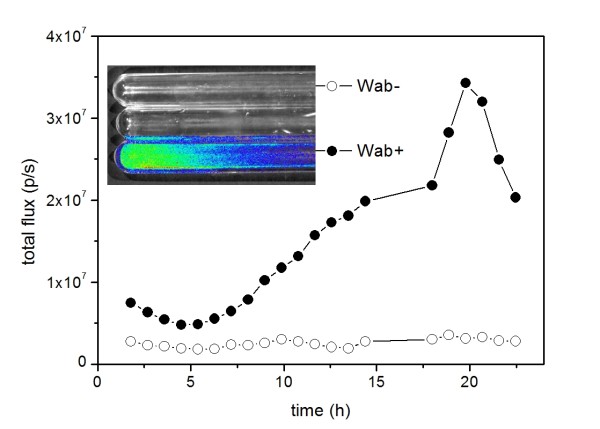
**On-line luminescence measurement of Wab+ and Wab- strains by In Vitro Imaging System (IVIS)**. Wab+ and Wab- cells were cultivated in static growth conditions as described in the text. With the system, real-time information about wax ester synthesis can be obtained directly from the cultivation without sampling. The inlet: Overlaid IVIS photo and luminescence data of the growth tubes at the end of the cultivation.

For monitoring and visualizing the WE biosynthesis on-line in static culture conditions without sampling, Xenogen In Vitro Imaging System (IVIS) was applied. Wab+ and Wab- were cultured in 10 ml of minimal salts medium supplemented with 110 mM glucose at room temperature for 23 h. For quantification of luminescent signal, overlaid photo and light-emission data was collected every hour with 10 min exposure time, reported as maximal radiance for a pixel within the growth tube. The pattern for signal curve was found to be similar compared to the previous experiment (Figure 3).

**Figure 4:**
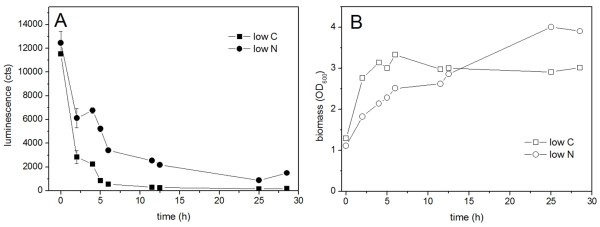
**Luminescence (A) and growth curves (B) of Wab+ cells grown under nitrogen limiting (low N) and carbon limiting (low C) conditions**.

**Figure 5:**
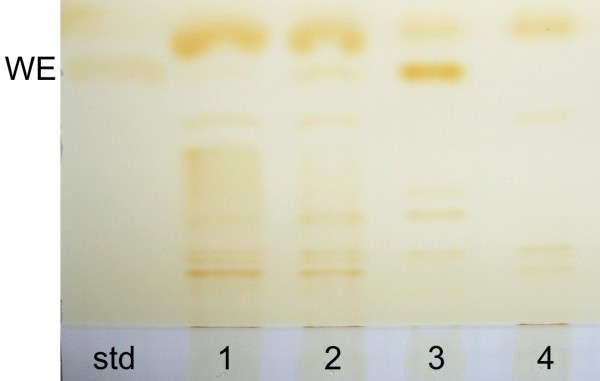
**Thin layer chromatography analyses of Wab+ cells grown under nitrogen limiting and carbon limiting conditions**. Cellular lipid compositions of Wab+ cells grown under nutrient limiting conditions for 28 h were analysed from extracted lipid samples proportioned to biomass. In the Figure: std - wax ester standard palmityol palmitate; 1 - Initial lipid composition of the cultivations; 2 - lipids extracted from control cultivation (grown in normal medium); 3 - lipids extracted from low nitrogen cultivation; 4 - lipids extracted from low carbon cultivation.

To study the performance of the monitoring system in conditions of carbon or nitrogen starvation, two-phased cultivations were carried out for the strain Wab+. In the first phase, the cells were cultivated for 16 h in normal MA/9 medium supplemented with 110 mM glucose. Thereafter, the cells were collected, divided in three fractions, and suspended to fresh mediums containing MA/9 salts and either 0.2% casein amino acids and 1 g/l NH_4_Cl (low C -medium), or 110 mM glucose and 0.1 g/l NH_4_Cl without casein amino acids (low N -medium). One third of the cells was suspended to normal MA/9 medium and considered as a control. The growth and luminescent signals are presented in Figure 4. The light emission of the low C cultivation decreased rapidly indicating that degradation and assimilation of the WE present in the cells does not interfere the monitoring of WE biosynthesis specifically. The degradation of WE was further verified by TLC analysis (Figure 5). In the end of the cultivation at 28 h, the presence of luminescent protein in the cells was confirmed by adding decanal, a LuxAB substrate, to the growth medium, producing prominent luminescent signal. The optical density slightly increased during the cultivation, from initial 1.3 to 3.0, which is due to the consumption of residual glucose (2 mM) and casein amino acids as carbon and energy sources. For the cultivation carried out in the low N -medium, the luminescent signal remained relatively high and declined more steadily compared to the low C cultivation. This could be expected since the WE accumulation is commonly related to high C/N ratio. The accumulation of WE was also shown by TLC analysis (Figure 5). However, the eventual decrease in luminescent signal was found to be related to the decrease in LuxAB content of the cells, which is probably due to the nitrogen limitation rather than decrease in the WE formation rate. This was also demonstrated by addition of decanal in the end of the cultivation, resulting in significantly lower luminescent signals compared to the low C cultivation (data not shown). In the beginning of the cultivation, the growth in low N cultivation was slower compared to the low C grown cells due to the adaptation for the nitrogen limiting conditions and lack of added amino acids, but eventually the cells reached a slightly higher biomass yield (optical density 3.9).

## Discussion

Bacterial biosynthesis of storage lipids such as WE is a dynamic process affected by several cellular and environmental factors. Studying the dynamics and optimizing such biochemical route is challenging since no specific and sensitive analysis methods are available for rapid detection of cellular metabolite levels. In this study, a proof of principle for studying bacterial wax ester metabolism on-line is introduced. The method is based on monitoring the cellular levels of specific intermediates of WE, fatty aldehydes, by utilizing reporter genes *luxAB*. For the study, a natural host for WE production, *A. baylyi *ADP1 expressing a bacterial luciferase LuxAB (strain Wab+), was exploited. In order to control and validate the monitoring system, a wax-negative knock-out strain of ADP1 expressing the same reporter system was constructed (Wab-).

The wax ester metabolism was monitored in batch cultivations in minimal salts medium supplemented with glucose as a carbon and energy source. Growth, carbon utilization, luminescence signal and relative WE content were monitored. For Wab+, correlation between luminescent signal and WE production rate was observed; according to NMR analysis, the relative amount of WE in the cells increased until approximately 34 h, after which the concentration slightly decreased during the hours 34 - 46. In the luminescence data, the signal increased during the exponential growth phase reaching the highest value at ~16 h, after which the signal significantly decreased and remained relatively constant until the end of the cultivation. Thus, the peak in luminescence data between 12 - 20 h can be speculated to represent the activation of WE route with enzymatic substrates being somewhat in imbalance so that more aldehyde is available for luminescence production. On the contrary, in the late exponential growth phase after 20 h the rate of aldehyde and assumingly WE production remained constant, and the enzymatic pathway seems to be more in steady-state. This can be partly explained with the role of WS/DGAT, the key enzyme of the final step in WE formation, which is known to be more active in the stationary growth phase, thus affecting the intermediate concentrations [13].

It was shown by the two-phased cultivations carried out with low carbon medium that the degradation of WE observed by the TLC analysis was not detected as luminescent signal. The luminescent protein presence and functionality was demonstrated by external long-chain aldehyde substrate addition in the end of the cultivation, neglecting the possibility of protein absence or inactivity. Thus, it is concluded that the degradation or assimilation of WE does not interfere the specific monitoring of WE synthesis, and on the other hand, the genetic construct is highly stable and functional even in carbon limited conditions. However, according to the TLC and NMR results of 34 h and 46 h time points of the normal medium cultivation, a decrease in WE content is observed, whereas the luminescent signal data at 34 - 46 h indicate the WE synthesis route being active in the stationary growth phase. This theory of continuous wax synthesis in stationary growth phase is also supported by literature [2] and the fact that glucose is steadily consumed. Thus, the monitoring system reveals an interesting fact of the WE metabolism: WE are produced constantly throughout the stationary growth phase but in the studied conditions they are more rapidly degraded than synthesized. Speculations related to the degradation and role of WE as a dynamic carbon and energy storage have been widely discussed by Fixter et al [2] and Finkelstein et al [14]. Furthermore, the role of WE in stationary growth phase of bacteria can be also seen in the growth patterns of Wab+ and Wab-; the strain Wab+ grows to slightly higher cell density.

Since the monitoring system presented here is a dynamic and biological system requiring cellular building blocks for protein production, the method was found to be unsuitable for lipid production studies on 'storage' conditions limiting the nitrogen availability. According to the results obtained from cultivations carried out in low N medium, nitrogen limitation strongly affected the protein maintenance and thus the luminescent signal. Still, compared to the low C grown cells, the signal was slightly higher during the cultivation indicating the response to wax synthesis. However, as the luciferase activity was determined in the end of the cultivation with decanal addition, significantly lower signal was obtained for low N cultivation compared to that of the low C cultivation despite the higher biomass yield of low N culture, indicating decrease in protein abundancy. As the genes *luxAB *were constitutively transcribed in the cell, it can be assumed that in conditions of sufficient supply of essential nutrients the luminescent protein availability is not a limiting factor for the analysis method.

The phenomenon was further established with on-line measurement exploiting IVIS to demonstrate the convenience of the method for real time measurements without the need for sampling. It was shown that the method is also applicable in static growth conditions which are natural for the *A. baylyi *strains prone to biofilm formation coupled with wax ester production. No significant differences were seen in the luminescent signal curve pattern compared to the cultivation applying normal sampling procedure.

As the monitoring system detects the WE intermediate, the method can be used for determining the changes in production rates, not the actual quantity or accumulation of WE in the cells. Thus, a parallel sensitive and specific detection method is still required to complete the method to be quantitative. However, the system provides valuable information about growth phase dependency, environmental factors affecting the production rates and induction, and differences between genetically modified strains. Also, this monitoring tool can be employed in searching new, as yet uncharacterized enzymes related to the wax ester pathway. Furthermore, as long chain aldehydes are crucial intermediates of bioenergy molecules produced by novel biosynthesis routes [15] we believe this method can have potentiality for direct applications in the field of metabolic engineering, synthetic biology and bioenergy production. Also, the method can potentially reveal novel cellular functions and roles of WE showing a highly dynamic character.

## Conclusions

This study introduces a proof of principle method for monitoring bacterial wax ester metabolism *in vivo *in real-time. The study demonstrates that by a simple gene insertion valuable information about wax ester metabolism can be obtained. The near future tasks involve more extensive and wide-ranging experimental set up to verify the usability and significance of this method for the research of lipids and other valuable long chain hydrocarbons in both wild-type and metabolically engineered production hosts.

## Methods

### Strains

The wild type strain *A. baylyi *ADP1 (DSM 24193) was used in the study. The single gene knock-out strain of ADP1 (gene deletion *acr1*, ACIAD3383) was kindly provided by Veronique de Berardinis (Genoscope, France). In the single gene knock-out mutant, the gene in question is replaced with a gene cassette containing a kanamycin resistance gene (*Kan^r^*) [16]. The strains were transformed with the construct i*luxAB_Cm^r ^*described in the section 'Genetic engineering'. *Escherichia coli *XL1-Blue (Stratagene, USA) transformed with i*luxAB_Cm^r^*/pAK400c was used as a control strain.

### Genetic engineering

The molecular work was carried out by using methods described by Sambrook *et al*. [17]. For digestions and ligations, the enzymes and buffers were provided by Fermentas (Lithuania) and used according to manufacturer's instructions. PCR reagents were provided by Finnzymes (Finland) (DNA polymerase PhusionTM and buffer) and Fermentas (nucleotides). Primers were ordered from ThermoFisher Scientific (USA) with appropriate restriction sites.

The natural transformation of ADP1 and ADP1Δ*acr1*:: *Kan^r^*/*tdk *was carried out by methodology described previously [11]. The colonies were selected on LA plates containing chloramphenicol (50 μg/ml) and glucose. The construct in the obtained strain was verified with colony PCR and further by sequencing. For *E. coli*, the transformation with the plasmid i*luxAB_Cm^r^*/pAK400c was carried out by electroporation as described earlier [17]. The colonies were selected on LA plates containing chloramphenicol (25 μg/ml) and glucose. The right construct was verified by restriction analysis. A synthetic gene cassette described by Santala *et al*. [11] was used as a scaffold for the construction of gene cassette i*luxAB_Cm^r ^*with flanking regions from neutral target knock-out gene *poxB *(ACIAD3381). The bacterial luciferace genes *luxAB *were amplified from the plasmid pCGLS1 [18] with primers ab39 (5'-ATATCATATGAAATTTGGAAACTTTTTGCTTAC-3') and ab40 (5'-ATATCTCGAGTTAGGTATATTCCATGTGGTACTTCTTAATATTATC-3') and inserted to the gene cassette using restriction sites *Nde*I and *Xho*I. The flanking region Gene_I' (downstream of *poxB*) was amplified from ADP1 genome with primers ab59 (5'-AATACCTAGGAGGTCAATTCTCCAGCTTTTTTATC-3') and ab60 (5'-AATAGGCCCCCGAGGCCTGTCAAAAGCATAGGAAGTGG-3') and cloned to the gene construct using restriction sites *Avr*II and *Sfi*I. The final gene cassette was amplified by PCR with primers ab57 (5'-ATATGGTACCCACACCAATTTTAGCACCCGGAAAAAATG-3') and ab60, the final product being 4112 bp long. Purification of the PCR products was carried out in every step using PCR purification kit (Fermentas) or gel extraction kit (Fermentas) for agarose gel. For *E. coli *transformation, the gene cassette i*luxAB_Cm^r ^*was cloned to the plasmid pAK400c [19] using restriction sites *Kpn*I and *Sfi*I.

### Medium composition

The minimal salts medium MA/9 described previously [11] was used for the cultivations. Cas amino acids (Difco, USA) were added at concentration (0.2 w-%) when appropriate. Glucose (110 mM) was used as a carbon and energy source. In carbon limiting medium, glucose was excluded from the medium components, and for nitrogen limiting medium, casein amino acids were excluded, respectively. Additionally, for nitrogen-limiting conditions 0.1 g/l was used instead of 1.0 g/l NH_4_Cl.

### Cultivations and luminescence determination

The batch cultivations for monitoring the WE metabolism in ADP1 was carried out in 60 ml medium/250 ml Erlenmeyer flasks in two parallel bottles. The strains were cultivated for 12 - 46 h in MA/9 medium at 30°C and 300 rpm. In the end of the cultivation the cells were collected by centrifugation (30 min, 30000 g). The batch cultivations for monitoring the effect of nitrogen and glucose starvation on WE metabolism were carried out in the same conditions for 16 h, after which the cells were collected by centrifugation (10 min, 30000 g) and the cells were inoculated in three mediums representing nitrogen-limiting, carbon-limiting and control cultivations. The cultivation was continued for additional 28 h in same conditions.

For luminescent measurement, samples were taken from the batch cultivations every 1 or 2 hours and 100 or 200 μl of a sample was applied on well plates in three parallel samples. The well plates were shaken for 2 minutes at 650 rpm and the luminescent signal was measured immediately with Victor 2 plate reader (Perkin Elmer Life Sciences, Finland). For LuxAB activity measurements, decanal (Sigma, USA) was added to the samples in final ratio of 1: 2000 before the measurement. Biomass determination was carried out by optical density measurement (OD_600_) at the wavelength 600 nm.

For visualizing and monitoring the WE synthesis in ADP1 on-line, cultivations inside the measuring chamber of Xenogen In Vitro Imaging System (IVIS^® ^Lumina, Caliper Life Sciences, USA) were carried out. The strains ADP1Δ*poxB*::i*luxAB_Cm^r ^*(Wab+) and ADP1Δ*poxB::*i*luxAB_Cm^r^Δacr1*::*Kan^r^*/*tdk*(Wab-) were cultivated in 10 ml of MA/9 medium with 2% glucose at room temperature without shaking for 23 h. For quantification of luminescent signal, overlaid photo and light-emission data was collected every hour with 10 min exposure time and the maximal radiance for a pixel within the growth tube was determined.

### Analysis for glucose consumption

Glucose consumption in the cultivations was measured with high performance liquid chromatography (HPLC). Culture samples were centrifuged at 20000 g for 5 minutes. Supernatant was collected and filtered through polycarbonate filter (Chromafil^® ^PET-45/25, Macherey-Nagel, Germany). The glucose concentration in the medium was determined with LC-20AC prominence liquid chromatograph (Shimadzu, USA) equipped with RID-10A refractive index detector, DGU-20A5 prominence degasser, CBM-20A prominence communications bus module, and SIL-20AC prominence autosampler. Shodex SUGAR SH1011 (Showa Denko KK, Japan) kept at 40°C was used as a column. Sulfuric acid (0.01 N) was used as an eluent at pumping rate of 0.6 ml/min. Identification and quantification of glucose was based on co-chromatography using an external standard. Mediums were used as controls.

### Lipid extraction

The lipids were extracted from the freeze-dried biomass samples with two parallel samples as described in Santala *et al*. [11] The amount of total lipids was determined gravimetrically. For TLC analyses, small-scale samples representing equal amounts of biomass were taken for extraction (1-3 ml). The samples were centrifuged at 20000 g for 5 min and the supernatant was discarded. The cells were suspended in 500 μl methanol and 250 μl of chloroform after which the tubes were shaken gently for an hour. The tubes were centrifuged at 20000 g for 5 min and lower phase (chloroform) was collected.

### WE visualization using TLC

In order to visualize the WE content in the cells, TLC analyses were carried out using 10 × 10 cm Silica Gel 60 F_254 _HPTLC glass plates with 2.5 × 10 cm concentrating zone (Merck, USA). Mobile phase used was n-hexane: diethyl ether: acetic acid 90: 15: 1. Palmityol-palmitate (Sigma) was used as a standard. Of extracted lipids, 30 μl of each sample was applied on the TLC plate and iodine was used for visualization.

### WE quantification using NMR

For quantitative determination of WE content in the cells, extracted total lipid fraction was analyzed using NMR. ^1^H NMR measurements were performed on Varian Mercury spectrometer (300 MHz). Samples (3 - 5.5 mg) were dissolved in 0.7 ml of chloroform-d_3 _(Sigma) and spectra were taken at ambient temperature. Trifluorotoluene was used as an internal standard (1.47 mg/0.7 ml of chloroform-d_3_). Chemical shifts were quoted as parts per million relative to tetramethylsilane (δ = 0) and spectra were processed using ACD NMR processor program. Phase correction and baseline correction were applied to all the spectra and the characteristic peaks were integrated. The areas of the peaks are directly proportional to the amount of each functional group. The number of protons in each group was also considered in the calculations. The peak at δ 4.05 ppm corresponds specifically to α-alkoxy methylene protons of alcohols in wax esters, whereas the signal at δ 3.65 ppm is produced by α-alkoxy methylene protons of free alcohols. Thus, the integral value of peak at δ 4.05 ppm was used to calculate the concentration of wax esters.

## Competing interests

The authors declare that they have no competing interests.

## Authors' contributions

SS and VS designed the study. SS performed the molecular work, microbiological work, and wrote the manuscript. SS and VS performed the metabolic analyses. EE carried out the NMR analyses and participated in manuscript drafting. VS and MK supervised and coordinated the study. All authors read and approved the final manuscript.
